# Cost-effectiveness of inactivated seasonal influenza vaccination in a cohort of Thai children ≤60 months of age

**DOI:** 10.1371/journal.pone.0183391

**Published:** 2017-08-24

**Authors:** Wanitchaya Kittikraisak, Piyarat Suntarattiwong, Darunee Ditsungnoen, Sarah E. Pallas, Taiwo O. Abimbola, Chonticha Klungthong, Stefan Fernandez, Suchada Srisarang, Tawee Chotpitayasunondh, Fatimah S. Dawood, Sonja J. Olsen, Kim A. Lindblade

**Affiliations:** 1 Influenza Program, Thailand Ministry of Public Health - U.S. Centers for Disease Control and Prevention Collaboration, Nonthaburi, Thailand; 2 Infectious Diseases Unit, Queen Sirikit National Institute of Child Health, Ministry of Public Health, Bangkok, Thailand; 3 Global Immunization Division, U.S. Centers for Disease Control and Prevention, Atlanta, Georgia, United States of America; 4 Laboratory of Virology, Armed Forces Research Institute of Medical Sciences, Bangkok, Thailand; 5 Influenza Division, U.S. Centers for Disease Control and Prevention, Atlanta, Georgia, United States of America; University of Washington, UNITED STATES

## Abstract

**Background:**

Vaccination is the best measure to prevent influenza. We conducted a cost-effectiveness evaluation of trivalent inactivated seasonal influenza vaccination, compared to no vaccination, in children ≤60 months of age participating in a prospective cohort study in Bangkok, Thailand.

**Methods:**

A static decision tree model was constructed to simulate the population of children in the cohort. Proportions of children with laboratory-confirmed influenza were derived from children followed weekly. The societal perspective and one-year analytic horizon were used for each influenza season; the model was repeated for three influenza seasons (2012–2014). Direct and indirect costs associated with influenza illness were collected and summed. Cost of the trivalent inactivated seasonal influenza vaccine (IIV3) including promotion, administration, and supervision cost was added for children who were vaccinated. Quality-adjusted life years (QALY), derived from literature, were used to quantify health outcomes. The incremental cost-effectiveness ratio (ICER) was calculated as the difference in the expected total costs between the vaccinated and unvaccinated groups divided by the difference in QALYs for both groups.

**Results:**

Compared to no vaccination, IIV3 vaccination among children ≤60 months in our cohort was not cost-effective in the introductory year (2012 season; 24,450 USD/QALY gained), highly cost-effective in the 2013 season (554 USD/QALY gained), and cost-effective in the 2014 season (16,200 USD/QALY gained).

**Conclusion:**

The cost-effectiveness of IIV3 vaccination among children participating in the cohort study varied by influenza season, with vaccine cost and proportion of high-risk children demonstrating the greatest influence in sensitivity analyses. Vaccinating children against influenza can be economically favorable depending on the maturity of the program, influenza vaccine performance, and target population.

## Background

Influenza results in a substantial disease burden worldwide and is costly to patients, employers, and society in terms of medical costs, lost wages, and lost productivity [1]. The most effective way to prevent illness and severe outcomes from influenza is through vaccination [2]. Influenza vaccines have been used since 1936 and are proven to be safe[3, 4]. The vaccine has been introduced into many national vaccination programs[4, 5]. In these countries, the World Health Organization (WHO) recommends national policy makers to take country-specific information about risk groups, disease burden, and cost-effectiveness into consideration to help shape the programs[6].

Since 2009, the Government of Thailand has annually purchased seasonal influenza vaccine for healthcare personnel and high-risk persons including children aged 6 months-2 years[7]. Each year, approximately 3.5 million doses of trivalent inactivated influenza vaccine (IIV3) are administered free of charge to the estimated 11 million high-risk persons on a first-come, first-served basis. In 2015, the Health Intervention and Technology Assessment Program of the Thai Ministry of Public Health published a cost-effectiveness analysis of a hypothetical annual influenza vaccination program of preschool- and school-aged children with IIV3 or trivalent live-attenuated influenza vaccine (LAIV)[8]. However, LAIV is not used routinely in Thailand, and healthy, school-aged children are not included among the recommended risk groups for influenza vaccination. To date, an economic evaluation of IIV3 among recommended risk groups administered through the national vaccination program has not been conducted in Thailand.

In this study, we determined the cost-effectiveness of IIV3 vaccination compared to no vaccination in children ≤60 months participating in a cohort study of influenza-associated illness in Bangkok, Thailand; cost was measured from a societal perspective using a one-year analytic horizon for each of three influenza seasons.

## Methods

### Setting

#### Pediatric Respiratory Infection Cohort Evaluation *(*PRICE study*)*

A prospective cohort study (Pediatric Respiratory Infection Cohort Evaluation, PRICE) to determine influenza burden among children living in the Bangkok Metropolitan areas who actively sought care (for any cause except acute respiratory illness) was established at the Queen Sirikit National Institute of Child Health (QSNICH; Bangkok)[9]. Enrollment occurred during August 2011-September 2013; healthy children aged 0–36 months were age- and time-matched with “high-risk” children defined as children with underlying conditions (asthma, prematurity, congenital heart disease, chronic lung or airway disease, abnormality of the upper airway, kidney disease, liver disease, neurologic/neuromuscular disease, hemoglobinopathy, metabolic disease, development delay immunosuppressive condition, and cancer). Enrolled children for whom matches could not be identified were allowed to participate in the study throughout the course of the study. Written parental informed consent was sought for all children. For two years during study follow-up, caregivers were contacted weekly to identify any acute respiratory illness (ARI) in the 1,149 enrolled children (659 healthy and 490 high-risk) and encouraged to bring children with ARI to the study clinic to have nasal and throat swabs collected and tested for influenza by polymerase chain reaction (PCR) at the Armed Forces Research Institute of Medical Sciences (Bangkok). In this study, the ARI was defined as presence of ≥2 of the following symptoms according to caregivers’ reports: fever, nasal discharge/congestion, cough, or sore throat. Of 3,458 ARI episodes identified, 3,078 (89%) were presented for care at QSNICH. Study staff interviewed caregivers 1–2 weeks following illness onset about illness management, outcome, and related expenses. Influenza vaccination status was verified using children’s vaccine cards. The study was approved by the ethics committees of the QSNICH, with the U.S. Centers for Disease Control and Prevention’s Institutional Review Board relied on the QSNICH’s determination. Eight laboratory-confirmed influenza cases (4.9%) were identified among vaccinated healthy children (H_vax_) in 2012, 9 cases (5.0%) in 2013, and 3 cases (3.8%) in 2014. Two influenza cases (1.9%) were identified among vaccinated high-risk children (HR_vax_) in 2012, 9 cases (6.4%) in 2013, and no cases (0%) in 2014. Thirty-three influenza cases (8.9%) were identified among unvaccinated healthy children (H_unvax_) in 2012, 35 cases (8.4%) in 2013, and 10 cases (4.7%) in 2014. Sixteen influenza cases (5.7%) were identified among unvaccinated high-risk children (HR_unvax_) in 2012, 19 cases (6.1%) in 2013, and 1 case (0.7%) in 2014. Length of illness (i.e., onset to illness resolution based on caregivers’ reports) in children with influenza were generally <12 days.

#### Influenza season and vaccination program in Thailand

Influenza viruses circulate year-round in Thailand with two peaks (June-November and January-March)[10]. The national influenza vaccination campaign runs annually during May-July. Therefore, we defined an influenza season as June through next May (e.g., the 2012 season was June 2012-May 2013)[11]. Influenza vaccine is not part of the routine Expanded Program on Immunization schedule which comprises the required vaccinations for children in Thailand; influenza vaccination is based on caregivers’ choice and was not administered as part of the PRICE study. Proportions of children vaccinated in the PRICE study are shown in Table 1. At their first vaccination opportunity, children aged <9 years are recommended two doses, ≥28 days apart, for full vaccination; one dose is recommended for subsequent years. Data from PRICE and other studies in Thailand show the Southern Hemisphere IIV3 was 64% effective against PCR-confirmed influenza virus infection in young children in 2012, 64% in 2013, and 24% in 2014 seasons[9, 12].

**Table 1:** Model parameters.

	Season			Source
	2012 (N = 918)	2013 (N = 1,046)	2014 (N = 516)	
**PROPORTION OF CHILDREN IN THE COHORT BY VACCINATION AND MEDICAL CONDITION**				
Vaccinated group				
Healthy	67%	56%	52%	PRICE
High-risk	33%	44%	48%	PRICE
Unvaccinated group				
Healthy	57%	57%	59%	PRICE
High-risk	43%	43%	41%	PRICE
**MEDIAN LENGTH OF ILLNESS IN DAYS**	**2012**	**2013**	**2014**	
Vaccinated group				
Healthy children, sick with influenza and treated in OPD	8.5	8	8	PRICE
Healthy children, sick with influenza and treated in IPD	9	0^b^	n/a^b^	PRICE
High-risk children, sick with influenza and treated in OPD	8.5	8.5	n/a^b^	PRICE
High-risk children, sick with influenza and treated in IPD	n/a ^b^	10.5	n/a^b^	PRICE
Unvaccinated group				
Healthy children, sick with influenza and treated in OPD	9	8	8	PRICE
Healthy children, sick with influenza and treated in IPD	10	6	21	PRICE
High-risk children, sick with influenza and treated in OPD	8	10	7	PRICE
High-risk children, sick with influenza and treated in IPD	10	12	n/a^b^	PRICE
**INFLUENZA VACCINATION**	**2012**	**2013**	**2014**	
Influenza vaccine uptake	29%	31%	29%	PRICE
Proportion of vaccinated children receiving 1 influenza vaccine dose	0.55	0.71	0.88	PRICE
Proportion of vaccinated children receiving 2 influenza vaccine doses	0.45	0.29	0.12	PRICE
**MEDIAN COST ASSOCIATED WITH INFLUENZA ILLNESS (USD)**^a^	**2012**	**2013**	**2014**	
Vaccinated group				
Healthy children, sick with influenza and treated in OPD	23.89	31.17	22.15	PRICE
Healthy children, sick with influenza and treated in IPD	286.14	n/a^b^	n/a^b^	PRICE
High-risk children, sick with influenza and treated in OPD	51.93	27.59	n/a^b^	PRICE
High-risk children, sick with influenza and treated in IPD	n/a^b^	483.68	n/a^b^	PRICE
Unvaccinated group				
Healthy children, sick with influenza and treated in OPD	27.97	23.13	30.83	PRICE
Healthy children, sick with influenza and treated in IPD	172.89	94.85	1,011.91	PRICE
High-risk children, sick with influenza and treated in OPD	18.97	21.82	18.77	PRICE
High-risk children, sick with influenza and treated in IPD	241.16	2,848.08	n/a^b^	PRICE
**COST OF INFLUENZA VACCINE ADMINISTRATION (USD/dose)**	**2012**	**2013**	**2014**	
Vaccine cost including storage and vaccine distribution for one IIV3 ready-to-use adult dose (cost includes half-dose administered to child and half-dose discarded)	3.64	3.68	3.48	NHSO
Cost for promotion, administration, and supervision	0.85	0.86	0.77	NHSO
**OUTCOMES**	**2012**	**2013**	**2014**	
Utility weight for healthy children	0.87			Canaway et al.[14]
Utility weight for children who were sick with influenza and treated in OPD	0.65			Perlroth et al.[15]
Utility weight for children who were sick with influenza and treated in IPD	0.50			Perlroth et al.
**NUMBER OF CHILDREN BECOMING SICK WITH INFLUENZA IN COHORT BY MODEL ARM**	**2012**	**2013**	**2014**	
Vaccinated group				
Healthy children, sick with influenza and treated in OPD	7	9	3	PRICE
Healthy children, sick with influenza and treated in IPD	1	0	0	PRICE
Healthy children, not sick with influenza	154	172	76	PRICE
High-risk children, sick with influenza and treated in OPD	2	7	0	PRICE
High-risk children, sick with influenza and treated in IPD	0	2	0	PRICE
High-risk children, not sick with influenza	103	132	73	PRICE
Unvaccinated group				
Healthy children, sick with influenza and treated in OPD	29	34	9	PRICE
Healthy children, sick with influenza and treated in IPD	4	1	1	PRICE
Healthy children, not sick with influenza	337	379	203	PRICE
High-risk children, sick with influenza and treated in OPD	13	17	1	PRICE
High-risk children, sick with influenza and treated in IPD	3	2	0	PRICE
High-risk children, not sick with influenza	265	291	150	PRICE

^a^Total cost of illness was a summation of direct medical, direct non-medical, and indirect or opportunity costs associated with influenza illness. The largest portion of costs among ill children in the cohort were direct costs with only a few cases incurring indirect or opportunity costs; vaccine administration cost was added for each vaccinated child.

^b^No cases

OPD: outpatient department; IPD: inpatient department; NHSO: National Health Security Office; PRICE; Pediatric Respiratory Infection Cohort Evaluation

### Decision-analytic model

**Fig 1:**
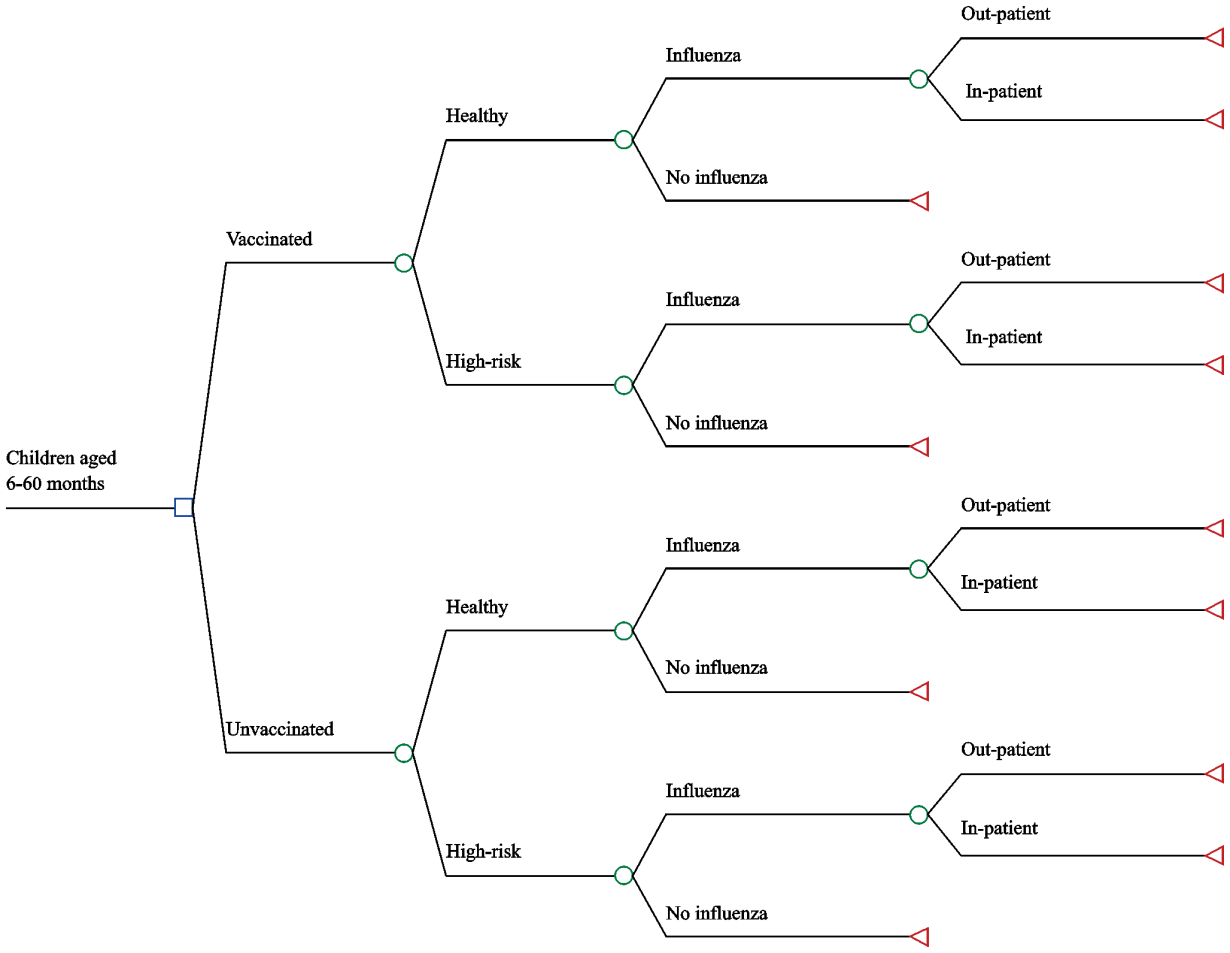
A schematic diagram representing the decision tree used in each year of the analysis.

To estimate the cost-effectiveness of IIV3 vaccination among children aged 6–60 months, a static decision tree model (Fig 1) was constructed to simulate the population of children in our cohort using TreeAge Pro Suite 2014 (Williamstown, MA). The proportions of children with influenza, treated in either outpatient (OPD) or inpatient departments (IPD), were derived from children followed weekly in the cohort (Table 1). Death was not considered in our model because there were no influenza-related deaths among enrolled children. Analysis was performed by influenza season (2012, 2013, and 2014) because influenza vaccine efficacy declines after 6–8 months[13].

### Perspective and analytic horizon

In this analysis, the societal perspective and one-year analytic horizon were used for each influenza season[16, 17]. Children included in each modeled influenza season were not mutually exclusive as they were followed for up to two years.

### Model parameters

#### Vaccination status and proportion of children by model arm

The proportions of healthy and high-risk children, children vaccinated, and influenza cases treated in OPD or IPD were taken from the PRICE study (Table 1). Results of statistical tests of differences across these populations are reported elsewhere[9].

#### Cost parameters

Direct costs included medical and diagnostic costs (paid out of pocket and by health insurance) and transportation costs. Indirect or opportunity costs were self-reported actual income loss or monetized value of productive work time lost related to care for ill children. Direct and indirect costs paid by caregivers were collected from post-illness interviews and the value of caregiver’s time was calculated using the human capital approach[18]. Caregivers were asked about days they missed work to care for ill children (if they worked), days the ill children missed daycare (if children went to daycare), and the caregivers’ average income per day. The indirect costs were calculated by multiplying days of missed work or daycare (whichever was higher) by the caregiver’s daily income. The time of caregivers who did not earn income was valued at the government-established minimum wage (300 Baht/day)[16, 17, 19]. Costs paid for by the health insurance system were from the QSNICH’s database. QSNICH is a public hospital; all medical costs are determined by government regulation except personnel charges paid by the government as salaries or overtime pay that were not charged to patients. Therefore, the personnel charges were not included in the analysis.

The annual cost of the vaccine program, obtained from the National Health Security Office (Nonthaburi, Thailand), included the cost of vaccine purchase, storage, transportation, promotional material, vaccine administration at local hospitals, and program supervision. The annual national program cost for these elements was divided by the number of doses purchased nationally in that year to derive cost per IIV3 ready-to-use adult dose. For the purpose of this analysis, the cost of one full IIV3 adult dose was applied for each child who received half of a ready-to-use adult dose with the other half discarded. The cost of the wasted half-dose was included in the analysis, but the costs of wastage more generally were not included as no official data on national wastage rates were available. Direct and indirect costs were converted from Thai Baht to US dollars (USD) using the relevant exchange rate (31.1, 30.7, 32.5 Baht to one USD in 2012, 2013, 2014, respectively)[20]. Median costs per influenza episode were calculated for healthy and high-risk children and vaccinated and unvaccinated groups and used in the model[21]. No discounting was performed because the analysis was conducted over a one-year analytic horizon. Spillover costs (e.g., costs associated with time healthcare providers spent vaccinating children or treating children with influenza and not performing other tasks) were not considered in the model. Median influenza-associated illness costs ranged from <20 USD in OPD to about 2,850 USD in IPD settings (Table 1).

#### Quality-adjusted life years and utility weights

Quality-adjusted life years (QALY) were used to quantify the health outcome of number of days ill with influenza. To determine the QALY value, the utility weight associated with one of three possible health states—well, ill with influenza requiring outpatient care, or ill with influenza requiring inpatient care—was multiplied by the fraction of a year lived in that state. For each of the four study groups (H_vax_, HR_vax_, H_unvax_, and HR_unvax_), the median number of days spent in each of the three health states was calculated from post-illness interview data. Utility weights (i.e., estimates of health-related quality of life based on how a sample of the general population would trade off years of full health to avoid years lived with various degrees of disability) for each of the three health states were drawn from published literature: 0.87 for well children [14], 0.65 for children mildly sick with influenza and treated in OPD, and 0.50 for children with more severe influenza illness requiring treatment in IPD[15]. For example, for the H_unvax_ group and the health state of influenza illness requiring inpatient care, the median number of days ill was 10 days, which translates to 0.86 QALY, i.e., [0.50*(10 days ill with influenza/365 days per year)+0.87*(355 days not ill with influenza/365 days per year)]. Spillover benefits (e.g., benefits associated with reduced events of related illnesses such as pneumonia or protecting other high-risk groups from influenza) were not considered in the model.

### Incremental cost-effectiveness ratio

Incremental cost-effectiveness ratios (ICERs) were calculated as the difference in the expected total costs between the vaccinated and unvaccinated groups divided by the difference in QALYs between groups. WHO threshold values were used as proxies for the decision maker’s willingness to pay to determine whether a public health intervention was cost-effective[22]. Compared to no vaccination, influenza vaccination was cost-saving if the ICER was negative, very cost-effective if the ICER was less than Thailand’s Gross Domestic Product per capita (GDPpc), cost-effective if the ICER was 1–3 times Thailand’s GDPpc, and not cost-effective if the ICER was >3 times Thailand’s GDPpc. The World Bank’s GDPpc values for Thailand for 2012, 2013, and 2014 (5,918 USD, 6,229 USD, and 5,977 USD, respectively) were used for comparing ICERs against these WHO thresholds[23].

### Sensitivity and scenario analyses

For each season modeled, univariate sensitivity analyses were conducted for vaccine cost (range: 0.5–4 times current price), rate of second IIV3 doses (range: 29–45%), proportion of high-risk children (range: 0–100%), utility weights (range: 0.73–0.95 for well children, 0.52–0.66 for children with influenza treated in OPD, and 0.05–0.58 for influenza treated in IPD) [15, 24–26], and proportion of influenza cases (range: 4.8–5% for healthy vaccinated children, 2.8–6.4% for high-risk vaccinated children, 7.3–8.9% for health unvaccinated children, 4.2–6.1% for high-risk unvaccinated children). Ranges were selected to reflect programmatically probable values and available data from the PRICE study or published literature. As utility weights were drawn from settings outside of Thailand, different combinations of weights from other literature sources and their averages were also used to calculate QALYs in multivariate sensitivity analyses. Scenario analyses were also conducted with alternative values for the proportion of high-risk children (10%, 5%) and cost of vaccine (2 and 3 times the current price) to project cost-effectiveness under implementation conditions beyond the PRICE cohort that might be encountered by the national influenza vaccine program.

## Results

### Base case

The expected total costs and QALYs were higher in vaccinated than unvaccinated children in all modelled seasons (i.e., vaccination was both more expensive and had more beneficial health outcomes compared to no vaccination). Compared to no vaccination, IIV3 vaccination among children aged 6–60 months in the PRICE study was not cost-effective in the 2012 season (ICER: 24,450 USD/QALY gained), highly cost-effective in the 2013 season (ICER: 554 USD/QALY gained), and cost-effective in the 2014 season (ICER: 16,200 USD/QALY gained; Table 2), based on WHO thresholds. In all seasons, the direct costs associated with healthcare services accounted for the majority of the costs of illness, while indirect costs associated with missed work or actual income loss of caregivers were minimal.

**Table 2:** Results of base case analysis comparing administration of seasonal trivalent inactivated influenza vaccination among children aged ≤60 months to no vaccination, 2012–2014.

	Expected value of total cost^a^ (USD)		Expected value of QALY^b^		ICER of vaccination to no vaccination (USD/QALY)	Interpretation based on WHO CEA thresholds[27]
	Vaccinated children	Unvaccinated children	Vaccinated children	Unvaccinated children		
2012 season	8.69 (vaccination costs: 6.49; OPD patient COI: 1.02; IPD patient COI: 1.18)	3.80 (OPD patient COI: 1.63; IPD patient COI: 2.18)	0.8698	0.8696	24,450	Not cost-effective
2013 season	10.31 (vaccination costs: 5.84; OPD patient COI: 1.47; IPD patient COI: 3.00)	9.59 (OPD patient COI: 1.60; IPD patient COI: 7.99)	0.8697	0.8684	554	Highly cost- effective
2014 season	5.22 (vaccination costs: 4.79; OPD patient COI: 0.43; IPD patient COI: N/A)	3.60 (OPD patient COI: 0.82; IPD patient COI: 2.78)	0.8699	0.8698	16,200	Cost-effective

^a^Expected value of total cost per child (rounded to the nearest US cent) is the product of the median cost of illness for each health outcome state (not ill with influenza, ill with influenza requiring outpatient care, ill with influenza requiring inpatient care) for each study group (vaccinated health children, vaccinated high-risk children, unvaccinated healthy children, unvaccinated high-risk children) multiplied by the proportion of children from the Pediatric Respiratory Infection Cohort Evaluation study who ended up in each health outcome state in each study group (i.e., the probability of any given child ending up in that health outcome state, given his or her study group assignment); for vaccinated children, the expected value of total cost also includes the cost of vaccine administration in addition to the cost of illness.

^b^The expected values of quality adjusted life years (rounded to four significant digits) are the products of the magnitude of each health outcome multiplied by the proportion of children in the Pediatric Respiratory Infection Cohort Evaluation study experiencing that health outcome for each study group and health outcome state.

Gross Domestic Product per capita: 5,918 USD (2012), 6,229 USD (2013), and 5,977 USD (2014)

QALY, quality adjusted life year; ICER, incremental cost-effective ratio; WHO, World Health Organization; CEA, cost-effectiveness analysis; OPD, outpatient department; COI, cost of illness; IPD, inpatient department

### Sensitivity analyses

**Fig 2:**
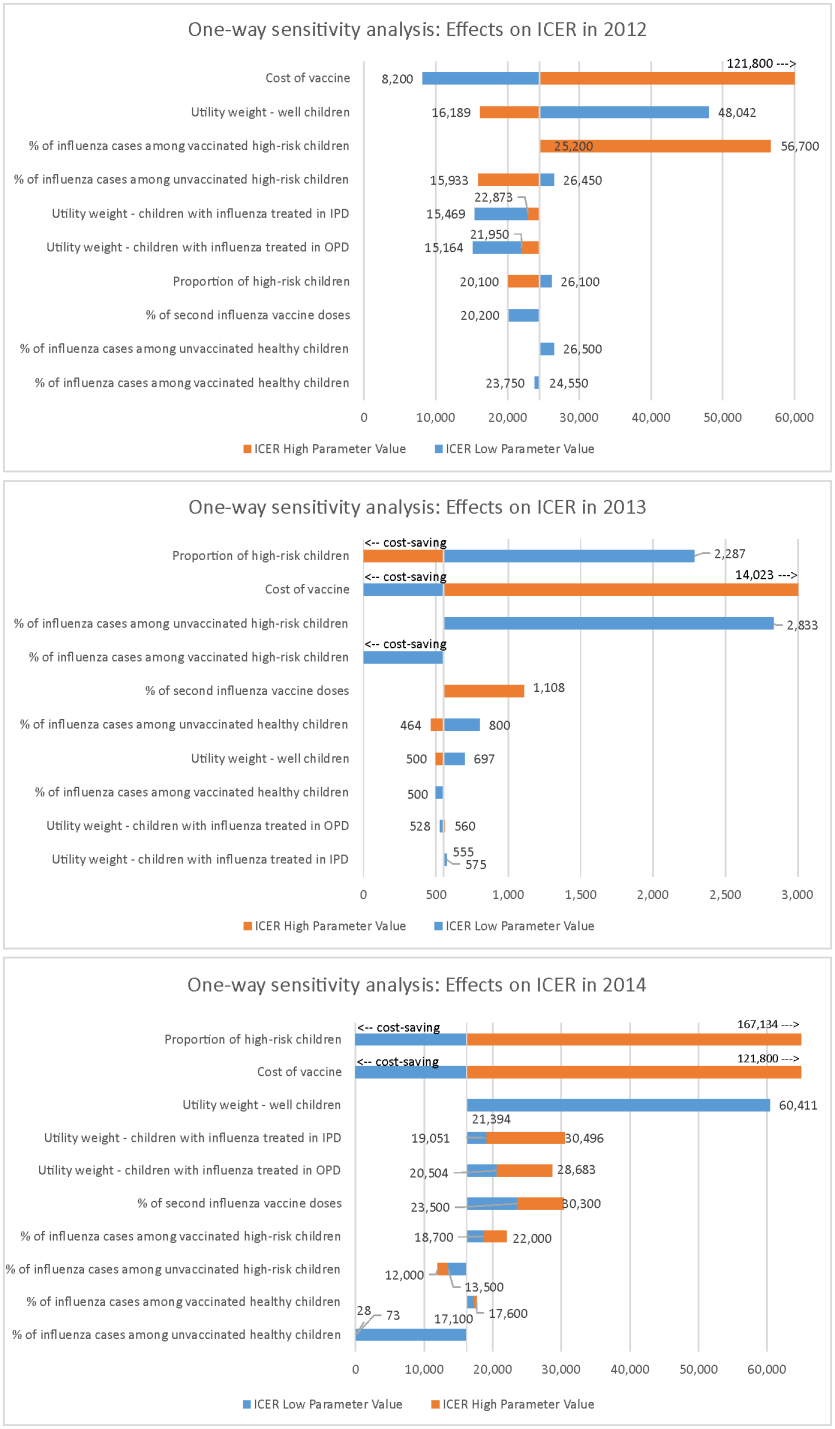
One-way sensitivity analyses. Incremental cost-effectiveness ratios are reported in US dollars per quality adjusted life year gained. Ranges of values used are as follows: Cost of vaccine, 0.5–4.0 times current vaccine cost; Utility weight—well children, 0.73–0.95; Utility weight—children with influenza treated in outpatient department, 0.52–0.66; Utility weight—children with influenza treated in inpatient department, 0.05–0.58; % of second influenza vaccine doses, 0.29–0.45; Proportion of high-risk children, 0–1; % of influenza cases among unvaccinated high-risk children, 4.2–6.1; % of influenza cases among unvaccinated healthy children, 7.3–8.9; % of influenza cases among vaccinated high-risk children, 2.8–6.4; and % of influenza cases among healthy children, 4.6–5.0. ICER: incremental cost-effectiveness ratio; OPD, outpatient department; IPD, inpatient department.

In univariate sensitivity analyses, the variables with the greatest influence on the ICER were vaccine cost (2012, 2013, 2014 seasons), utility weight for children without influenza illness (2012 and 2014 seasons), rate of influenza cases among high-risk vaccinated and unvaccinated children (2012 and 2013 seasons), and proportion of high-risk children (2013 and 2014 seasons) (Fig 2 and S1 Table). In 2012, IIV3 vaccination became cost-effective in all multivariate sensitivity analyses of combinations of utility weights, except when utility weights of 0.92 was used for well children, 0.659 for children with influenza treated in OPD, and 0.514 for children treated in IPD (S2 Table). In 2013, regardless of the choice of utility weights, IIV3 vaccination remained highly cost-effective by WHO thresholds compared to no vaccination. In contrast, mixed results were observed in 2014 with cost-effectiveness strongly influenced by the choice of utility weight. In 2012, IIV3 vaccination became cost-effective only when the vaccine price was reduced by half (S2 Table). IIV3 vaccination was not cost-effective in 2012 regardless of threshold set for high-risk children (0%-100%). IIV3 became cost-effective (cost-saving to highly cost-effective) in 2013 at all thresholds set for high-risk children (0%-100%). However, IIV3 was only cost effective (cost-saving to highly cost-effective) only when there was no high-risk children (0%) or only a small percentage of high-risk children (5%). When the vaccine price was changed, IIV3 vaccination was cost-saving if the price was reduced by half in both 2013 and 2014 and remained either highly cost-effective or cost-effective if the price was increased by ≥2 times. Taking all modelled seasons into account, the most favorable scenario in terms of cost-effectiveness was when vaccine price was reduced by half. The least favorable scenarios were when the population consisted of 100**%** high-risk children and when vaccine price increased to ≥2 times the current price.

## Discussion

Compared to no vaccination, we found that the cost-effectiveness of IIV3 vaccination among children participating in the PRICE study varied by influenza season. Cost/QALY gained was lowest in the 2013 season (i.e., most cost-effective) and highest in the 2012 season (i.e., least cost-effective). Our results were consistent across varying utility weights for the 2012 and 2013 seasons, but not for 2014. In scenario analyses, we found that cost-effectiveness differed depending on the target population for vaccination and the vaccine price.

The findings from the 2012 model resemble the introduction of an influenza vaccine program where the program bears start-up costs, administering two vaccine doses to each child. Many children in our study required two vaccine doses in 2012 for full vaccination as it was their first eligible vaccination opportunity although the models for subsequent years also accounted for the cohort’s new members who required two vaccine doses. As a result, the vaccine cost in 2012 was higher than subsequent years. As a vaccination program matures, as illustrated by the 2013 and 2014 models, a greater return on investment may be realized depending largely on how well the vaccines perform and the number of newly eligible children requiring two vaccine doses for those particular seasons. In 2013, vaccine effectiveness (VE) was 64% [9] and the intervention was considered highly cost-effective based on ICER thresholds. In 2014, VE was 24% due to a poor match between the circulating and vaccine strains [12], but the intervention was still cost-effective due to the lower cost resulting from fewer doses required to fully vaccinate cohort children. New members entered the cohort only when they sought care at the study clinic (a children’s hospital without a maternity ward, and therefore without systematic recruitment of newborns); the share of children requiring two doses of IIV3 in 2013 and 2014 is therefore lower in our analysis than would be expected in a hypothetical national influenza vaccination program for children aged 6 months-2 years in which roughly half of each year’s birth cohort would be newly eligible each year.

From a programmatic standpoint, risk group prioritization plays an important role in the performance of a vaccine program. Our findings suggest that in a hypothetical population consisting of 100**%** of healthy children or 5**%** or 10**%** of high-risk children, but with the same rate of influenza as observed in the PRICE study, the vaccination program would be generally cost-effective or cost-saving after an introductory year, due to the higher incidence of influenza in healthy than in high-risk children observed in the cohort[21]. Notably, in a year with an effective vaccine, the program was also cost-saving when vaccinating only high-risk children because of the reduced illness duration among vaccinated children. In our cohort, the incidence of influenza was much higher in healthy than in high-risk children, possibly because they might have had greater social contact with others (e.g., attending daycare) compared to high-risk children who tended to stay at home. With limited vaccine supply, our findings underscore the need to take vaccination target population composition and disease severity and illness duration into account for vaccine prioritization to maximize health benefits per administered vaccine dose.

Other studies, mostly conducted in developed countries, have examined the cost-effectiveness of an influenza vaccine among children. Although one previous modeling study examined influenza vaccination scenarios for children over two years old in Thailand [8], our study assessed the cost-effectiveness of vaccinating younger children against seasonal influenza illness using IIV3 based on empirical cost and outcome data from a cohort study over three influenza seasons. Our findings are consistent with those previously reported from other countries that influenza vaccination among young children is typically economically advantageous from a societal perspective[28–32]. The findings highlight that performance of an influenza vaccine program is closely linked with VE, which varies from year to year depending on the match of circulating viruses to vaccine strains. Thus, annual program evaluations to assess vaccine coverage in target groups and VE are needed for accurate cost evaluations.

Our analysis has a few limitations. High-risk children in the PRICE study were oversampled and may not reflect the true proportion of high-risk children in the general population, which was thought to be <10%. We assumed all vaccinated children received the vaccine from the government and that the standard government payment schedule accurately captured the hospital’s production costs. The observed vaccine wastage rate was high **(**50**%)** due to the lack of ready-to-use pediatric doses in country, resulting in children receiving half of a ready-to-use adult dose with the other half discarded; while costs of this PRICE study-specific wastage were included in the analysis (resulting in higher vaccination costs and therefore less favorable ICERs for vaccination), overall national wastage costs were not included due to lack of official data (although internal NHSO estimates put such wastage at approximately 5% and therefore of lesser influence than the study-specific wastage on the vaccination strategy costs). All children with ARI in the study were invited to the hospital including those with mild symptoms who might not require a hospital visit; therefore, the total costs may be higher than what would be observed in other health care settings. Results are season-specific and should not be generalized to other influenza seasons. Lastly, our static model, though easy to interpret and similar to those used to evaluate the cost-effectiveness of influenza vaccination in other settings [33–36], did not account for the variation and distribution of input parameters (e.g., vaccination rate, length of illness, cost) that likely exists for the general population of children in Thailand beyond the PRICE study and may be sensitive to small cell sizes. This static model also did not account for indirect benefits of childhood influenza immunization (e.g., reduced influenza transmission in the general population, resulting reductions in influenza cases among other at-risk populations such as the elderly. Lastly, our study did not capture the side effects that may occur as a result of vaccination.

In summary, our results suggest that vaccinating young children against influenza can be economically favorable from a societal standpoint depending on the maturity of the program, influenza vaccine performance, and target population. Our findings presented as dollars per QALY gained allows policymakers to compare the cost-effectiveness of influenza vaccination of children with other potential investments in preventive health services.

## Supporting information

S1 Table Parameters used in one-way sensitivity analyses. (XLSX)

S2 Table Results of one-way sensitivity analysis and scenario analysis comparing administration of seasonal trivalent inactivated influenza vaccine among children aged ≤60 months to no vaccination, 2012–2014. ^a^Expected value of total cost per child (rounded to the nearest US cent) is the product of the median cost of illness for each health outcome state (not ill with influenza, ill with influenza requiring outpatient care, ill with influenza requiring inpatient care) for each study group (vaccinated healthy children, vaccinated high-risk children, unvaccinated healthy children, unvaccinated high-risk children) multiplied by the proportion of children from the PRICE study who ended up in each health outcome state in each study group (i.e., the probability of any given child ending up in that health outcome state, given his or her study group assignment); for vaccinated children, the expected value of total cost also includes the cost of vaccine administration in addition to the cost of illness. ^b^The expected values of QALYs (rounded to four significant digits) are the products of the magnitude of each health outcome multiplied by the proportion of children in the PRICE study experiencing that health outcome for each study group and health outcome state.^c^Children with underlying medical condition e.g., prematurity, congenital heart disease, chronic lung or airway disease, neuromuscular disease. High-risk children in the PRICE cohort were oversampled to about 40% of the entire sample. ^d^Difference at >4 significant digits. Gross Domestic Product per capita: 5,918 USD **(**2012**)**, 6,229 USD **(**2013**)**, and 5,977 USD **(**2014**)**. QALY, quality-adjusted life year; ICER, incremental cost-effectiveness ratio; WHO, World Health Organization; CEA, cost-effectiveness analysis. Sensitivity 1: 0.95 for healthy, 0.58 for influenza treated in outpatient department, and 0.58 for influenza treated in inpatient department (Tarride et al.)[24]. Sensitivity 2: 0.87 for healthy 0.52 for influenza treated in outpatient department, and 0.05 for influenza treated in inpatient department (Perlroth et al.)[15]. Sensitivity 3: 0.93 for healthy, 0.558 for influenza treated in outpatient department and inpatient department (Luce et al.)[25]. Sensitivity 4: 0.92 for healthy, 0.659 for influenza treated in outpatient department, and 0.514 for influenza treated in inpatient department (Lee et al.)[26]. Sensitivity 5: 0.91 for healthy, 0.59 for influenza treated in outpatient department, and 0.44 for influenza treated in inpatient department (average of all utility weights used).
(DOCX)

S1 File Dataset used for the analysis. (XLSX)
