# Supplementary material for: Cost-effectiveness of inactivated seasonal influenza vaccination in a cohort of Thai children ≤60 months of age
Source: PLoS One. 2017 Aug 24;12(8):e0183391. doi: 10.1371/journal.pone.0183391 (PMC5570265; doi:10.1371/journal.pone.0183391)
Supplement: S2 Table — aExpected value of total cost per child (rounded to the nearest US cent) is the product of the median cost of illness for each health outcome state (not ill with influenza, ill with influenza requiring outpatient care, ill with influenza requiring inpatient care) for each study group (vaccinated healthy children, vaccinated high-risk children, unvaccinated healthy children, unvaccinated high-risk children) multiplied by the proportion of children from the PRICE study who ended up in each health outcome state in each study group (i.e., the probability of any given child ending up in that health outcome state, given his or her study group assignment); for vaccinated children, the expected value of total cost also includes the cost of vaccine administration in addition to the cost of illness. bThe expected values of QALYs (rounded to four significant digits) are the products of the magnitude of each health outcome multiplied by the proportion of children in the PRICE study experiencing that health outcome for each study group and health outcome state.cChildren with underlying medical condition e.g., prematurity, congenital heart disease, chronic lung or airway disease, neuromuscular disease. High-risk children in the PRICE cohort were oversampled to about 40% of the entire sample. dDifference at >4 significant digits. Gross Domestic Product per capita: 5,918 USD (2012), 6,229 USD (2013), and 5,977 USD (2014). QALY, quality-adjusted life year; ICER, incremental cost-effectiveness ratio; WHO, World Health Organization; CEA, cost-effectiveness analysis. Sensitivity 1: 0.95 for healthy, 0.58 for influenza treated in outpatient department, and 0.58 for influenza treated in inpatient department (Tarride et al.)[24]. Sensitivity 2: 0.87 for healthy 0.52 for influenza treated in outpatient department, and 0.05 for influenza treated in inpatient department (Perlroth et al.)[15]. Sensitivity 3: 0.93 for healthy, 0.558 for influenza treated in outpatient department and inpatient [file pone.0183391.s002.docx]

**S2 Table. Results of One-way Sensitivity Analysis and Scenario Analysis Comparing Administration of Seasonal Trivalent Inactivated Influenza Vaccine among Children Aged ≤60 Months to No Vaccination, 2012-2014**

1. **One-way sensitivity analysis**

|  | Expected value of total cost^a^ (USD) | | Expected value of QALY^b^ | | ICER of vaccination to no vaccination **(**USD/QALY**)** | Interpretation based on WHO CEA thresholds [27] |
| --- | --- | --- | --- | --- | --- | --- |
|  | Vaccinated children | Unvaccinated children | Vaccinated children | Unvaccinated children |  |  |
| **2012 season** | | | | | | |
| Base case | 8.69 | 3.80 | 0.8698 | 0.8696 | 24,450 | Not cost-effective |
| Sensitivity 1 | 8.69 | 3.80 | 0.9497 | 0.9493 | 12,209 | Cost-effective |
| Sensitivity 2 | 8.69 | 3.80 | 0.8696 | 0.8692 | 12,209 | Cost-effective |
| Sensitivity 3 | 8.69 | 3.80 | 0.9327 | 0.9323 | 12,209 | Cost-effective |
| Sensitivity 4 | 8.69 | 3.80 | 0.9197 | 0.9195 | 24,450 | Not cost-effective |
| Sensitivity 5 | 8.69 | 3.80 | 0.9083 | 0.9081 | 24,450 | Not cost-effective |
| **2013 season** | | | | | | |
| Base case | 10.31 | 9.59 | 0.8697 | 0.8684 | 554 | Highly cost-effective |
| Sensitivity 1 | 10.31 | 9.59 | 0.9495 | 0.9480 | 480 | Highly cost-effective |
| Sensitivity 2 | 10.31 | 9.59 | 0.8695 | 0.8681 | 514 | Highly cost-effective |
| Sensitivity 3 | 10.31 | 9.59 | 0.9325 | 0.9311 | 514 | Highly cost-effective |
| Sensitivity 4 | 10.31 | 9.59 | 0.9196 | 0.9183 | 554 | Highly cost-effective |
| Sensitivity 5 | 10.31 | 9.59 | 0.9079 | 0.9067 | 641 | Highly cost-effective |
| **2014 season** | | | | | | |
| Base case | 5.22 | 3.60 | 0.8699 | 0.8698 | 16,200 | Cost-effective |
| Sensitivity 1 | 5.22 | 3.60 | 0.9498 | 0.9498 | 20,250 | Not cost-effective |
| Sensitivity 2 | 5.22 | 3.60 | 0.8698 | 0.8697 | 16,200 | Cost-effective |
| Sensitivity 3 | 5.22 | 3.60 | 0.9328 | 0.9328 | 18,000 | Not cost-effective |
| Sensitivity 4 | 5.22 | 3.60 | 0.9199 | 0.9198 | 16,200 | Cost-effective |
| Sensitivity 5 | 5.22 | 3.60 | 0.9085 | 0.9067 | 944 | Highly cost-effective |

1. **Scenario analysis**

|  | Expected value of total cost^a^ (USD) | | Expected value of QALY^b^ | | ICER of vaccination to no vaccination **(**USD/QALY**)** | Interpretation based on WHO CEA thresholds [27] |
| --- | --- | --- | --- | --- | --- | --- |
|  | Vaccinated children | Unvaccinated children | Vaccinated children | Unvaccinated children |  |  |
| **Scenario 1: 100% high-risk children**^c^ | | | | | | |
| 2012 base case | 7.48 | 3.46 | 0.8699 | 0.8697 | 20,100 | Not cost-effective |
| 2013 base case | 14.06 | 19.56 | 0.8696^d^ | 0.8696^d^ | Dominant | Cost saving |
| 2014 base case | 4.79 | 0.12 | 0.8700^d^ | 0.8700^d^ | 167,134 | Not cost-effective |
| **Scenario 2: 10% high-risk children** | | | | | | |
| 2012 base case | 9.11 | 4.01 | 0.8697 | 0.8695 | 25,500 | Not cost-effective |
| 2013 base case | 8.05 | 3.87 | 0.8697 | 0.8677 | 2,090 | Highly cost- effective |
| 2014 base case | 5.53 | 5.46 | 0.8698 ^γ^ | 0.8698 ^γ^ | 1,258 | Highly cost-effective |
| **Scenario 3: 5% high-risk children** | | | | | | |
| 2012 base case | 9.20 | 4.04 | 0.8697 | 0.8695 | 25,800 | Not cost- effective |
| 2013 base case | 7.72 | 3.00 | 0.8698 | 0.8676 | 2,145 | Highly cost- effective |
| 2014 base case | 5.57 | 5.76 | 0.8698 ^γ^ | 0.8698 ^γ^ | Dominant | Cost saving |
| **Scenario 4: 0% high-risk children** | | | | | | |
| 2012 base case | 9.29 | 4.07 | 0.8697 | 0.8695 | 26,100 | Not cost-effective |
| 2013 base case | 7.39 | 2.13 | 0.8698 | 0.8675 | 2,287 | Highly cost- effective |
| 2014 base case | 5.61 | 6.06 | 0.8698^d^ | 0.8698^d^ | Dominant | Cost saving |
| **Scenario 5: vaccine cost halved** | | | | | | |
| 2012 base case | 5.44 | 3.80 | 0.8698 | 0.8696 | 8,200 | Highly cost-effective |
| 2013 base case | 7.39 | 9.59x | 0.8697 | 0.8684 | Dominant | Cost saving |
| 2014 base case | 2.82 | 3.60 | 0.8699 | 0.8698 | Dominant | Cost saving |
| **Scenario 6: vaccine cost doubled** | | | | | | |
| 2012 base case | 15.18 | 3.80 | 0.8698 | 0.8696 | 56,900 | Not cost-effective |
| 2013 base case | 16.15 | 9.59 | 0.8697 | 0.8684 | 5,046 | Highly cost- effective |
| 2014 base case | 10.01 | 3.60 | 0.8699 | 0.8698 | 64,100 | Not cost-effective |
| **Scenario 7: vaccine cost tripled** | | | | | | |
| 2012 base case | 21.67 | 3.80 | 0.8698 | 0.8696 | 89,350 | Not cost- effective |
| 2013 base case | 21.98 | 9.59 | 0.8697 | 0.8684 | 9,531 | Cost-effective |
| 2014 base case | 14.81 | 3.60 | 0.8699 | 0.8698 | 112,100 | Not cost- effective |
| **Scenario 8: vaccine cost quadrupled** | | | | | | |
| 2012 base case | 28.16 | 3.80 | 0.8698 | 0.8696 | 121,800 | Not cost-effective |
| 2013 base case | 27.82 | 9.59 | 0.8697 | 0.8684 | 14,023 | Cost-effective |
| 2014 base case | 19.60 | 3.60 | 0.8699 | 0.8698 | 160,000 | Not cost-effective |

^a^Expected value of total cost per child (rounded to the nearest US cent) is the product of the median cost of illness for each health outcome state (not ill with influenza, ill with influenza requiring outpatient care, ill with influenza requiring inpatient care) for each study group (vaccinated healthy children, vaccinated high risk children, unvaccinated healthy children, unvaccinated high risk children) multiplied by the proportion of children from the PRICE study who ended up in each health outcome state in each study group (i.e., the probability of any given child ending up in that health outcome state, given his or her study group assignment); for vaccinated children, the expected value of total cost also includes the cost of vaccine administration in addition to the cost of illness.

^b^The expected values of QALYs (rounded to four significant digits) are the products of the magnitude of each health outcome multiplied by the proportion of children in the PRICE study experiencing that health outcome for each study group and health outcome state.

^c^Children with underlying medical condition e.g., prematurity, congenital heart disease, chronic lung or airway disease, neuromuscular disease. High-risk children in the PRICE cohort were oversampled to about 40% of the entire sample.

^d^Difference at >4 significant digits.

Gross Domestic Product per capita**:** 5,918 USD **(**2012**)**, 6,229 USD **(**2013**)**, and 5,977 USD **(**2014**)**

QALY, quality-adjusted life year; ICER, incremental cost-effectiveness ratio; WHO, World Health Organization; CEA, cost-effectiveness analysis

Sensitivity 1: 0.95 for healthy, 0.58 for influenza treated in outpatient department, and 0.58 for influenza treated in inpatient department (Tarride et al.)[24]

Sensitivity 2: 0.87 for healthy 0.52 for influenza treated in outpatient department, and 0.05 for influenza treated in inpatient department (Perlroth et al.)[15]

Sensitivity 3: 0.93 for healthy, 0.558 for influenza treated in outpatient department and inpatient department (Luce et al.)[25]

Sensitivity 4: 0.92 for healthy, 0.659 for influenza treated in outpatient department, and 0.514 for influenza treated in inpatient department (Lee et al.)[26]

Sensitivity 5: 0.91 for healthy, 0.59 for influenza treated in outpatient department, and 0.44 for influenza treated in inpatient department (average of all utility weights used)
